# Individual participant data meta-analysis with mixed-effects transformation models

**DOI:** 10.1093/biostatistics/kxab045

**Published:** 2021-12-30

**Authors:** Bálint Tamási, Michael Crowther, Milo Alan Puhan, Ewout W Steyerberg, Torsten Hothorn

**Affiliations:** Institut für Epidemiologie, Biostatistik und Prävention, Departement Biostatistik, Universität Zürich, Hirschengraben 84, CH-8001 Zürich, Switzerland; Department of Medical Epidemiology and Biostatistics, Karolinska Institutet, 171 77 Stockholm, Sweden; Institut für Epidemiologie, Biostatistik und Prävention, Departement Epidemiologie, Universität Zürich, Hirschengraben 84, CH-8001 Zürich, Switzerland; Department of Biomedical Data Sciences, Leiden University Medical Center, Albinusdreef 2, 2333 ZA Leiden, the Netherlands; Institut für Epidemiologie, Biostatistik und Prävention, Departement Biostatistik, Universität Zürich, Hirschengraben 84, CH-8001 Zürich, Switzerland

**Keywords:** Individual participant data, Meta-analysis, Mixed-effects model, Prognostic modeling, Regression, Time-to-event outcomes, Transformation model

## Abstract

One-stage meta-analysis of individual participant data (IPD) poses several statistical and computational challenges. For time-to-event outcomes, the approach requires the estimation of complicated nonlinear mixed-effects models that are flexible enough to realistically capture the most important characteristics of the IPD. We present a model class that incorporates general normally distributed random effects into linear transformation models. We discuss extensions to model between-study heterogeneity in baseline risks and covariate effects and also relax the assumption of proportional hazards. Within the proposed framework, data with arbitrary random censoring patterns can be handled. The accompanying $\textsf{R}$ package **tramME** utilizes the Laplace approximation and automatic differentiation to perform efficient maximum likelihood estimation and inference in mixed-effects transformation models. We compare several variants of our model to predict the survival of patients with chronic obstructive pulmonary disease using a large data set of prognostic studies. Finally, a simulation study is presented that verifies the correctness of the implementation and highlights its efficiency compared to an alternative approach.

## 1. Introduction

In evidence synthesis, individual participant data (IPD) meta-analysis has several advantages over traditional meta-analytic approaches based on aggregated study-level statistics. Using individual-level data allows detailed data checking and transformations, verification and harmonization of variables and model parameterizations, as well as the application of more appropriate analytic methods, which in turn improve both the quality of the data and the analysis.

Stewart *and others* (2012) compare one-stage and two-stage IPD meta-analytic approaches, that is, analyzing the data in one joint model or aggregating study-level information and combining these aggregated statistics using conventional meta-analytic methods, respectively. They highlight that one of the main advantages of a one-stage approach is that it allows the various modeling assumptions to be formulated precisely and explicitly. Although Burke *and others* (2017) note that, when all methodological details are handled properly, one-stage and two-stages techniques should give similar results, one-stage IPD meta-analysis leads to increased power, handles missing observations more naturally, and takes the correlations among model parameters properly into account. The main disadvantages of a one-stage approach are the increased complexity of the statistical model and the potential computational issues with the estimation.

Although many applications focus on synthesizing findings of intervention studies, IPD meta-analysis can also be used in prognostic and diagnostic settings. Debray *and others* (2015) compare the main differences between IPD meta-analysis in intervention research and prognostic/diagnostic studies in their aims, approaches, and challenges. Debray *and others* (2013) demonstrate the importance of investigating the degree of variation of prognostic effects across studies and point out that using average effects when the heterogeneity is substantial, can be detrimental to the prediction performance.

The focus here is to develop a statistical model based on the mixed-effects extension of the transformation model framework by Hothorn *and others* (2018) tailored to one-stage IPD meta-analysis of a collection of prognostic studies. Specifically, we apply our model on a large-scale data set of prognostic studies focusing on survival prediction of patients with chronic obstructive pulmonary disease (COPD), provided by the COPD Cohorts Collaborative International Assessment (3CIA,, Soriano *and others*, 2015). We assess the study-level heterogeneity in the effects of the prognostic factors on the survival of COPD patients. Additionally, we derive individual-level predictions and compare different variants of our model based on their predictive performances. Extensions of the “global” prediction model, that is, a fixed-effects setting, with a single set of coefficients of the prognostic factors that is expected to apply broadly (see Steyerberg, 2019, Chapter 14), are considered. Our approach is capable of incorporating the most relevant features of the data for IPD meta-analysis, namely, study-level clustering of observations, heterogeneous effect sizes, time-to-event outcomes with interval censoring, and the possible presence of non-proportional hazards.

## 2. IPD meta-analysis with mixed-effects models

Combining data of individuals from different studies, which investigate different populations and have distinctive study characteristics, leads to clusters of correlated observations. A correct statistical model to analyze such data should account for the correlation structure of the grouped observations in order to draw valid inferences. Mixed-effects models provide a theoretically attractive approach to model the distribution of the outcome of interest conditionally on common study-specific characteristics in a one-stage IPD meta-analysis (Debray *and others*, 2013).

The heterogeneity in the effects of covariates across various studies can also be assessed and properly accounted for using mixed-effects models in an IPD meta-analysis (Tudur Smith *and others*, 2005; Steyerberg *and others*, 2019). Capturing the various sources of variability correctly is especially important in prognostic modeling, where external validity of the prognostic model is of primary concern (Steyerberg, 2019). In mixed-effects models, multivariate random effects can represent study-level heterogeneity in baseline risks as well as in the prognostic factor effects.

The estimation of regression models for time-to-event outcomes introduces specific challenges. First, the presence of various forms of censoring is typical in such settings. Although interval-censoring, due to periodic follow-up of the patients’ status, is very common in practice, most of the currently available software implementations of mixed-effects models either only allow for random right-censoring or provide limited random-effects capability in the case of interval-censored observations (Rondeau *and others*, 2012; Therneau, 2020).

There are differences in how various regression models for time-to-event outcomes handle the baseline hazard rates of the study population. The most popular approach is the Cox proportional hazards model—and its mixed-effects or “frailty” extensions—, which treats the baseline hazards as nuisance parameters and maximize the partial likelihood. However, in certain situations, for example, when the primary interest is to predict the *absolute risks* for the individual participants, models that aim to describe the full survival distributions are more appealing. Flexible parametric survival models have been proposed that approximate the baseline hazards by using spline functions either on the log-hazard scale (Crowther and Lambert, 2014) or on the log-cumulative hazard scale (Royston and Parmar, 2002; Crowther *and others*, 2014).

Another important aspect of IPD meta-analysis of time-to-event outcomes is how to account for heterogeneities in the baseline risks of different study populations. Prognostic models for IPD should allow for between-study differences in the outcome distributions, for the same values of the prognostic factors. A common approach of adjusting for baseline differences is by stratifying for studies, that is, by estimating a model with separate baseline hazard functions for each study but with common covariate effects in a proportional hazards setting (Michiels *and others*, 2005; Tudur Smith *and others*, 2005). Although this approach provides great flexibility in accounting for heterogeneities in unobserved characteristics, the estimation of large number of parameters can pose computational challenges. For this reason, several authors (Tudur Smith *and others*, 2005) considered models with frailty terms, that is, random effects that capture individual baseline characteristics, under the assumption of *proportionality* to a common baseline risk.

Violations of the proportional hazards (PH) assumption are common in meta-analyses of survival outcomes. A common way of relaxing the PH assumption is to allow for time-varying effects of the covariates. The time-dependent hazard or cumulative hazard ratios can then be estimated, for example, parametrically by using spline basis functions (Royston and Parmar, 2002; Crowther *and others*, 2014).

The validation of prognostic models is an integral part of the model-building process. As Steyerberg and Harrell (2016) emphasize, the evaluation of a prognostic model should address its *external validity*. In the context of prognostic models based on IPD from multiple studies, an “internal–external” validation scheme can be applied to gauge the predictive performance of the model on previously unseen data from a new study (Royston *and others*, 2004).

The aim of this article is to introduce the class of mixed-effects transformation models for individual participant data meta-analysis, and to provide an assessment of the approach in a prognostic setting based on data from multiple studies. The random effects account for the correlation structure among the observations in the same study. The heterogeneity in baseline risks as well as in the effects of the covariates are captured either by stratification or by including multivariate random effects. Moreover, within this model class, randomly censored observations can be naturally integrated under various forms of censoring. Greater flexibility may be achieved by introducing time-varying effects for the covariates. The likelihood-based estimation and inference can be carried out in the $\textsf{R}$ add-on package **tramME** (Tamási and Hothorn, 2021a). The estimation procedure is based on the Laplace approximation of the log-likelihood function, and the gradients are calculated with automatic differentiation (Kristensen *and others*, 2016), which makes our approach very efficient in terms of computational speed compared to alternatives.

## 3. Methods

### 3.1. Mixed-effects transformation models

Our general statistical framework for one-stage IPD meta-analysis with time-to-event outcomes is motivated as an extension of *linear transformation models* (Box and Cox, 1964; Cheng *and others*, 1995) with multivariate random effects. The transformation functions are estimated from the data using the specific parameterization proposed by Hothorn *and others* (2018). In its general form, the regression model for $i=1, 2, \dots, I$ studies, $j=1, 2, \dots, n_{i}$ participants in study $i$, with $q$ prognostic factors, ${\boldsymbol{x}}_{ij}$, and individual survival time outcomes denoted by $t_{ij}$ can be written as

(3.1) \begin{align*} {\mathbb{P}}(T_{ij} \leq t_{ij} \mid {\boldsymbol{X}}_{ij} = {\boldsymbol{x}}_{ij}, {\boldsymbol{\mathcal{B}}}_{i} = {\boldsymbol{b}}_{i})&= 1 - \exp\left[-\exp\left\{h_{i}(t_{ij} \mid {\boldsymbol{x}}_{ij} )\right\} \right] \label{eq:mod_p} \\ \end{align*}

(3.2) \begin{align*} h_{i}(t_{ij} \mid {\boldsymbol{x}}_{ij} ) &= h_{i}(t_{ij}) + {\boldsymbol{x}}_{ij}^{\top}({\boldsymbol{\beta}}(t_{ij}) + {\boldsymbol{b}}_{i}), \label{eq:mod_h} \end{align*}

with normal random effects ${\boldsymbol{\mathcal{B}}}_{i} \sim {\mathcal{N}}_{q}({\mathbf{0}}, {\boldsymbol{\Sigma}}{})$. The transformation model of the conditional distribution of the outcome in (3.1) is defined by an inverse link function and a conditional transformation function $h_i$. In our specific application, the *inverse link function* is set to the cumulative distribution function of the *minimum extreme value distribution*, that is, $F(z)=1-\exp(-\exp(z))$, which leads to the time-varying extension of the *proportional hazards* model. The choice of the inverse link function corresponds to parameterizing the log-cumulative hazard function $h_i$. In an alternative approach for a similar setting, Garcia *and others* (2019) use the distribution function of the standard logistic distribution to arrive at a time-varying extension of the proportional odds model. In general, all real distribution functions with log-concave density are applicable as inverse link functions (Hothorn *and others*, 2018). McLain and Ghosh (2013) discuss that the proportional hazards model and the proportional odds model represent two special cases in a continuum of transformation models with individual-level gamma-distributed frailty. In theory, it is possible to estimate the frailty parameter of this term, but in practice it is often unfeasible (Aalen *and others*, 2008, Section 6.4.4).

In (3.2), we split up the conditional transformation function ($h_i$) into two parts: the first part, $h_{i}(t_{ij})$, captures the study-specific *baseline risk* and the second part, ${\boldsymbol{x}}_{ij}^{\top}({\boldsymbol{\beta}}(t_{ij}) + {\boldsymbol{b}}_{i})$, models the—possibly time-varying—study-specific effects. In this latter part, we already introduced several assumptions: First, the effects of the covariates are additive to the baseline risks on the transformation scale. Second, at a given survival time ($t_{ij}$), the effects are linear, and third, the random coefficients (aka “random slopes”) of the covariates (${\boldsymbol{b}}_{i}$) are time-independent. In certain cases, the random slopes are omitted, which corresponds to assuming homogeneity in the effects across studies. In the models presented in Section 4, we do not assume a specific structure for the covariance matrix of the random effects (${\boldsymbol{\Sigma}}{}$), which means that we estimate $q (q+1) / 2$ variance–covariance parameters.

The study-level *baseline transformation function* ($h_{i}(t_{ij})$), is equal to the log-cumulative baseline hazard when the inverse link is the minimum extreme value distribution function. Following the parameterization proposed by Hothorn *and others* (2018), the baseline transformation and the time-varying fixed effects ${\boldsymbol{\beta}}(t_{ij})$, are approximated with general smooth functions as $h(t_{ij}) = {\boldsymbol{a}}(t_{ij})^{\top}{\boldsymbol{\vartheta}}_{0}$, with the help of a basis function ${\boldsymbol{a}}()$ and the corresponding vector of parameters ${\boldsymbol{\vartheta}}_{0}$. Section A.1 of the Supplementary material available at *Biostatistics* online provides more information on this basis. The approach of approximating the baseline transformation function with flexible parametric methods is conceptually similar to the model by Royston and Parmar (2002) or the approach by Liu *and others* (2016, 2017), with some important differences in the computational details. Our parameterization relies on polynomials in Bernstein form, as first suggested by McLain and Ghosh (2013).

The different specifications for IPD meta-analysis examined in this study represent further simplifications of the fairly general model described in (3.1) and (3.2). The general, study-specific transformation functions can be simplified as $h_{i}(t_{ij}) = h(t_{ij}) + a_{i}$ with the extended vector of random effects, $\left(\mathcal{A}_{i}, {\boldsymbol{\mathcal{B}}}_{i}^{\top}\right)^{\top} = {\boldsymbol{\mathcal{B}}}^{\star}_{i} \sim {\mathcal{N}}_{q+1}({\mathbf{0}}, {\boldsymbol{\Sigma}}{}^{\star})$, that is assumed to be the sum of a common baseline risk function and an additive (on the transformation scale), normally distributed random frailty term. Additionally, time-independent fixed effects of the covariates can be assumed ${\boldsymbol{\beta}}(t_{ij}) = {\boldsymbol{\beta}}$, and we can even exclude the random slopes from our model.

Table 1 summarizes the various versions of the model defined by (3.1) and (3.2) that we estimate in Section 4. These models differ in whether they assume PH for the covariate effects, in how they handle the heterogeneity of the baseline risks, and whether they allow for study-level variability of the prognostic factor effects.

**Table 1.** Alternative specifications of the mixed-effects transformation model for IPD meta-analysis.

	**Specification**	**Properties**
**Model 1**	${\mathbb{P}}\left(T_{ij} \leq t_{ij} \mid {\boldsymbol{X}}_{ij} = {\boldsymbol{x}}_{ij}, {\boldsymbol{\mathcal{B}}}^{\star}_{i} = (a_{i}, {\boldsymbol{b}}_{i}^{\top})^{\top}\right) = F\left(h(t_{ij}) + a_{i} + {\boldsymbol{x}}_{ij}^{\top}({\boldsymbol{\beta}} + {\boldsymbol{b}}_{i}) \right),$ ${\boldsymbol{\mathcal{B}}}^{\star}_{i} \sim {\mathcal{N}}_{q+1}({\mathbf{0}}, {\boldsymbol{\Sigma}}{}^{\star})$	• Proportional baseline risks • Proportional hazards • Random slopes
**Model 2**	${\mathbb{P}}\left(T_{ij} \leq t_{ij} \mid {\boldsymbol{X}}_{ij} = {\boldsymbol{x}}_{ij}, {\boldsymbol{\mathcal{B}}}_{i} = {\boldsymbol{b}}_{i}\right) = F\left(h_{i}(t_{ij}) + {\boldsymbol{x}}_{ij}^{\top}({\boldsymbol{\beta}} + {\boldsymbol{b}}_{i}) \right),$ ${\boldsymbol{\mathcal{B}}}_{i} \sim {\mathcal{N}}_{q}({\mathbf{0}}, {\boldsymbol{\Sigma}}{})$	• Stratified baseline risks • Proportional hazards • Random slopes
**Model 3**	${\mathbb{P}}\left(T_{ij} \leq t_{ij} \mid {\boldsymbol{X}}_{ij} = {\boldsymbol{x}}_{ij}, {\boldsymbol{\mathcal{B}}}^{\star}_{i} = (a_{i}, {\boldsymbol{b}}_{i}^{\top})^{\top}\right) = F\left(h(t_{ij}) + a_{i} + {\boldsymbol{x}}_{ij}^{\top}({\boldsymbol{\beta}}(t_{ij}) + {\boldsymbol{b}}_{i}) \right),$ ${\boldsymbol{\mathcal{B}}}^{\star}_{i} \sim {\mathcal{N}}_{q+1}({\mathbf{0}}, {\boldsymbol{\Sigma}}{}^{\star})$	• Proportional baseline risks • Nonproportional hazards • Random slopes
**Model 4**	${\mathbb{P}}\left(T_{ij} \leq t_{ij} \mid {\boldsymbol{X}}_{ij} = {\boldsymbol{x}}_{ij}, {\boldsymbol{\mathcal{B}}}_{i} = {\boldsymbol{b}}_{i}\right) = F\left(h_{i}(t_{ij}) + {\boldsymbol{x}}_{ij}^{\top}({\boldsymbol{\beta}}(t_{ij}) + {\boldsymbol{b}}_{i}) \right),$ ${\boldsymbol{\mathcal{B}}}_{i} \sim {\mathcal{N}}_{q}({\mathbf{0}}, {\boldsymbol{\Sigma}}{})$	• Stratified baseline risks • Nonproportional hazards • Random slopes
**Model 5**	${\mathbb{P}}\left(T_{ij} \leq t_{ij} \mid {\boldsymbol{X}}_{ij} = {\boldsymbol{x}}_{ij}\right) = F\left(h_{i}(t_{ij}) + {\boldsymbol{x}}_{ij}^{\top}{\boldsymbol{\beta}} \right)$	• Stratified baseline risks • Proportional hazards • Fixed effects only
**Model 6**	${\mathbb{P}}\left(T_{ij} \leq t_{ij} \mid {\boldsymbol{X}}_{ij} = {\boldsymbol{x}}_{ij}\right) = F\left(h_{i}(t_{ij}) + {\boldsymbol{x}}_{ij}^{\top}{\boldsymbol{\beta}}(t_{ij}) \right)$	• Stratified baseline risks • Nonproportional hazards • Fixed effects only

Models 1 and 2 are PH mixed-effects specifications, where the effects of the prognostic factors are assumed to be constant over time. Moreover, Model 1 captures the differences among study-level baseline risks using a random frailty term, proportional to a common baseline cumulative hazard function, whereas Model 2 estimates a separate function for each study in the data set.

Models 3 and 4 include time-varying fixed effects of the covariates, approximated with the use basis transformations (see Section A.1 of the Supplementary material available at *Biostatistics* online). These specifications relax the PH assumption of the previous two variants. Similar to Model 1, Model 3 assumes proportionality in the baseline risks and introduces an additional random intercept. By contrast, Model 4, just as Model 2, is stratified for studies for estimating baseline differences in a more flexible way.

Specifications Models 5 and 6 represent a fixed-effects only approach of IPD meta-analysis, to be compared with the more complex mixed-effects models. These models can be regarded as fully parametric versions of the Cox regression, both stratified for studies to capture differences in baseline risks. Model 6 allows non-PH, by including time-varying effects.

### 3.2. Estimation

The parameter vector ${\boldsymbol{\vartheta}} = \left({\boldsymbol{\vartheta}}_{0}^{\top}, {\boldsymbol{\beta}}^{\top}\right)^{\top}$ collects the fixed-effects parameters (either fixed over time or time-varying, approximated with the help of basis transformations), denoted by ${\boldsymbol{\beta}}$, and the coefficients of the basis approximation of the (possibly stratified) baseline transformation function, ${\boldsymbol{\vartheta}}_{0}$. Moreover, the vector of unobservable random effects (random slopes and intercepts, wherever they are necessary) is denoted by ${\boldsymbol{b}}_{i}$; its normal distribution is parameterized by ${\boldsymbol{\Sigma}}{}$. We can write then the likelihood function of the mixed-effects transformation models as

(3.3) \begin{align*} {\boldsymbol{\vartheta}}\left({\boldsymbol{\vartheta}}, {\boldsymbol{\Sigma}}{}\right) &= \prod_{i=1}^{I}\int_{{\boldsymbol{b}}_{i} \in {\mathbb{R}}^{q}}{\boldsymbol{\vartheta}}_{i}({\boldsymbol{\vartheta}}, {\boldsymbol{\Sigma}}{}, {\boldsymbol{b}}_{i})\mathsf{\,d}{\boldsymbol{b}}_{i} = \prod_{i=1}^{I}\int_{{\boldsymbol{b}}_{i} \in {\mathbb{R}}^{q}}\prod_{j=1}^{n_{i}}{\boldsymbol{\vartheta}}_{ij}({\boldsymbol{\vartheta}} \mid {\boldsymbol{b}}_{i})\phi({\boldsymbol{b}}_{i};{\boldsymbol{\Sigma}}{})\mathsf{\,d}{\boldsymbol{b}}_{i}, \label{eq:lik} \end{align*}

where ${\boldsymbol{\vartheta}}_{i}({\boldsymbol{\vartheta}}, {\boldsymbol{\Sigma}}{}, {\boldsymbol{b}}_{i})$ stands for the joint likelihood function of study $i$, and ${\boldsymbol{\vartheta}}_{ij}({\boldsymbol{\vartheta}} \mid {\boldsymbol{b}}_{i})$ is the *conditional* likelihood contribution of a participant $j$ in study $i$, while $\phi({\boldsymbol{b}}_{i};{\boldsymbol{\Sigma}}{})$ is the multivariate density function of ${\mathcal{N}}_{q}({\mathbf{0}}, {\boldsymbol{\Sigma}}{})$. The observations are assumed to be conditionally independent. The likelihood functions of the fixed effects only specifications (Models 5 and 6) are given in Hothorn *and others* (2018).

Because the mixed-effects transformation model introduced above parameterizes the conditional distribution function of the outcome directly, the likelihood contributions of the individual observations (survival times, defined on $t_{ij} \in [0, \infty)$) for *exactly observed* event times are

(3.4) \begin{align*} \label{eq:lik_contrib_exact} {\boldsymbol{\vartheta}}_{ij}({\boldsymbol{\vartheta}} \mid {\boldsymbol{b}}_{i}) &= f(h_{i}(t_{ij}) + {\boldsymbol{x}}_{ij}^{\top}({\boldsymbol{\beta}}(t_{ij}) + {\boldsymbol{b}}_{i})) (h_{i}^{\prime}(t_{ij}) + {\boldsymbol{x}}_{ij}^{\top}{\boldsymbol{\beta}}^{\prime}(t_{ij})),\end{align*}

where $f$, $h^{\prime}, {\boldsymbol{\beta}}^{\prime}$ are the derivative functions of $F$, $h$, ${\boldsymbol{\beta}}$, respectively. Under random censoring, the likelihood contributions can be expressed as

(3.5) \begin{align*} \small \label{eq:lik_contrib_cens} {\boldsymbol{\vartheta}}_{ij}({\boldsymbol{\vartheta}} \mid {\boldsymbol{b}}_{i}) &= \left\{ \begin{array}{lll} 1 - F(h_{i}(\underline{t}_{ij}) + {\boldsymbol{x}}_{ij}^{\top}({\boldsymbol{\beta}}(\underline{t}_{ij}) + {\boldsymbol{b}}_{i})) & y \in (\underline{t}_{ij}, \infty) & ``\text{right censored}'' \\[1em] F(h_{i}(\bar{t}_{ij}) + {\boldsymbol{x}}_{ij}^{\top}({\boldsymbol{\beta}}(\bar{t}_{ij}) + {\boldsymbol{b}}_{i})) & y \in (0, \bar{t}_{ij}] & ``\text{left censored}'' \\[1em] \begin{aligned} & F(h_{i}(\bar{t}_{ij}) + {\boldsymbol{x}}_{ij}^{\top}({\boldsymbol{\beta}}(\bar{t}_{ij}) + {\boldsymbol{b}}_{i})) \\ & \qquad - F(h_{i}(\underline{t}_{ij}) + {\boldsymbol{x}}_{ij}^{\top}({\boldsymbol{\beta}}(\underline{t}_{ij}) + {\boldsymbol{b}}_{i})) \end{aligned} & y \in (\underline{t}_{ij}, \bar{t}_{ij}] & ``\text{interval censored}''. \\[1em] \end{array} \right.\end{align*}

The maximum likelihood estimates of ${\boldsymbol{\vartheta}}$ and ${\boldsymbol{\Sigma}}{}$ are obtained by maximizing the natural logarithm of (3.3),

(3.6) \begin{align*} \ell({\boldsymbol{\vartheta}}, {\boldsymbol{\Sigma}}{}) &= \sum_{i=1}^{I}\log \int_{{\boldsymbol{b}}_{i} \in {\mathbb{R}}^{q}}{\boldsymbol{\vartheta}}_{i}({\boldsymbol{\vartheta}}, {\boldsymbol{\Sigma}}{}, {\boldsymbol{b}}_{i})\mathsf{\,d}{\boldsymbol{b}}_{i},\label{eq:loglik} \end{align*}

with a set of linear constraints that ensure that $h_{i}(t_{ij}\mid {\boldsymbol{x}}_{ij})$ are monotonically increasing in $t_{ij}$, to guarantee in turn that the conditional distribution functions are also nondecreasing (see Hothorn *and others*, 2018).

There are several ways of numerically evaluating the multivariate integral in the log-likelihood function (Pinheiro and Bates, 1995). The adaptive Gauss–Hermite quadrature gives accurate approximations with large enough number of quadrature points but becomes infeasible when the dimension of the random-effect vector is large. In our approach, the integral in (3.6) is approximated with Laplace’s method (see Kristensen *and others*, 2016, and Section A.1 of the Supplementary material available at *Biostatistics* online), which can be efficiently implemented even for high-dimensional problems.

Automatic differentiation is used, in conjunction with the Laplace approximation, to efficiently calculate the exact derivatives of the log-likelihood function (Skaug, 2002). Our approach builds on the Template Model Builder (TMB, Kristensen *and others*, 2016), which implements automatic differentiation and Laplace approximation to perform the maximum likelihood estimation of non-linear latent variable models. Asymptotic inference can be undertaken utilizing the automatic gradients and the Hessian of the log-likelihood function (Tamási and Hothorn, 2021a).

## 4. Application: IPD meta-analysis of prognostic studies of COPD patients

### 4.1. Description of the study

The large-scale data set provided by the COPD Cohorts Collaborative International Assessment (3CIA,, Soriano *and others*, 2015) contains 17 843 patients (with 4852 deaths and 91 576 person-years of follow-up) from a set of 25 cohort studies. The cohorts are very heterogeneous concerning patient characteristics, geographic location, study sizes, and observed events. Soriano *and others* (2015) provide additional information on the search protocol and inclusion/exclusion criteria of the studies in the data set. Bellou *and others* (2019) conducted a formal systematic review of prognostic models for COPD outcomes. Using the PROBAST methodology (Wolff *and others*, 2019), they identified several prognostic models for COPD mortality prediction as low risk of bias. All of these models were, at least in part, developed or validated on subsets of the 3CIA data set.

The outcome of interest is the time to death of patients with COPD. In several cohorts of the data set, as the patients were followed up monthly, the event-times were rounded to whole months. This kind of inexactness in event times, that is, time of death only can be ascertained up to an interval of months, is very common in practice. Although it is done very rarely in applications, the statistical approach should address this *interval-censored* nature of the outcome.

Of the available prognostic factors in the 3CIA data set, we used three covariates for our modeling purposes: age, ${FEV_{1}}$, and the modified Medical Research Council (mMRC) dyspnea score. The choice of focusing on this subset of predictors in the 3CIA data set is motivated by previous results that demonstrated their use in similar settings (Puhan *and others*, 2012). The covariates are well defined and their measurement is standardized, and, as a result, they are routinely included in various prognostic indices (Bellou *and others*, 2019). Consequently, these variables were recorded in almost every COPD cohort. This in turn reduces the risk of problems stemming from inconsistent variable definitions among studies. Section B of the Supplementary material available at *Biostatistics* online presents properties of the data (distribution, missingness, censoring) and explores the between-study heterogeneity in the 3CIA data set.

### 4.2. IPD meta-analysis with transformation models

The six models specified in Table 1 are estimated on the 3CIA data set using maximum likelihood (see Section 3). The log-likelihood values of the various specifications are listed in Table 2. As the results show, the versions with stratified baseline risks, that is, a separate log-cumulative baseline hazard function estimated for each cohort, result in improved model fits at the expense of considerably larger number of parameters. Moreover, including random prognostic factor effects and time-varying fixed effects also increase the value of the log-likelihood.

**Table 2.** Summary measures of in-sample fit, model complexity, and out-of-sample predictive performance of the six model variants. $\sum_{i}\ell_{i}^{\text{C-OOS}}$ denotes the aggregated centered out-of-sample log-likelihood values. The last column shows the order of the models in terms of out-of-sample predictive performance

	Number of parameters	Log-likelihood	$\sum_{i}\ell_{i}^{\text{C-OOS}}$	Rank
Model 1	18	$-$ 27 552.10	1269.16	3
Model 2	124	$-$ 27 380.48	1277.59	2
Model 3	30	$-$ 27 533.83	1219.42	4
Model 4	136	$-$ 27 370.06	1289.83	1
Model 5	118	$-$ 27 444.18	1180.41	6
Model 6	130	$-$ 27 415.24	1199.27	5

**Fig. 1.**
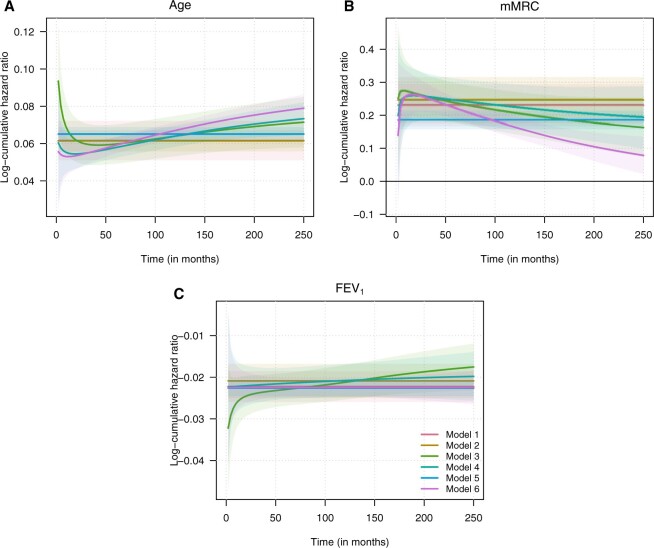
Conditional mean effects of the prognostic factors and their 95% pointwise confidence intervals.

The estimated values of the mean prognostic factor effects are similar across the six model specifications. Figure 1 compares the estimates of the fixed effects from the six models, along with their 95% pointwise confidence intervals, plotted against time. According to the results, all three prognostic factors have significant effects on the survival of COPD patients on the presented time interval. By construction Models 1, 2, and 5 have time-constant effects, while the other three specifications allow the (conditional) mean effect sizes to vary over time. Although the models that allow for non-PH indicate some degree of time-dependence in the prognostic factor effects, especially in the case of age and mMRC, these deviations are, in general, mild and the various models seem to agree on the magnitudes of the effect sizes.

From the results of the mixed-effects models for IPD meta-analysis, we can calculate *predictive intervals* for the prognostic factor effects. These intervals provide ranges for effect sizes to be expected for a new, yet unseen, study. We can approximate these intervals using the asymptotic distribution of the maximum likelihood estimates as $\beta(t)_{k} \sim {\mathcal{N}}\left(\hat\beta(t)_{k}, \hat\omega^{2}_{\hat\beta(t)_{k}} + \hat\sigma^{2}_{k}\right)$, where $\hat\beta(t)_{k}$ denotes the maximum likelihood estimate of the (possibly time-varying) effect of covariate $k$, $\hat\omega^{2}_{\hat\beta(t)_{k}}$ is its variance, which is calculated from the asymptotic distribution of the raw model parameters using the delta method, and $\hat\sigma^{2}_{k}$ stands for the estimated variance of the corresponding random slope. It should be noted that this approach ignores the uncertainty about the within-study and between-study estimates of the variability in the covariate effects, hence the true finite-sample predictive distribution is likely to have heavier tails than the normal approximation.

**Fig. 2.**
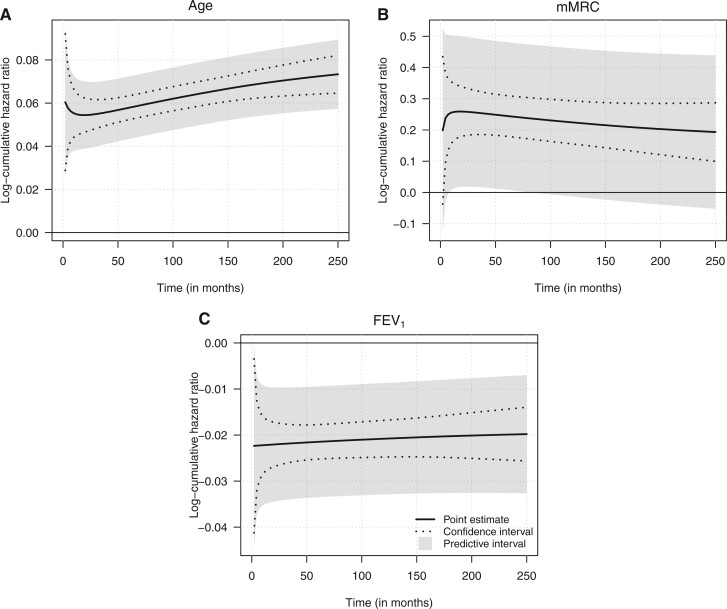
Model 4: 95% pointwise confidence intervals and predictive intervals of the prognostic factor effects on the log-cumulative hazard scale.

Figure 2 compares the prediction and confidence intervals for the prognostic factors estimated with Model 4. The differences between the prediction intervals and the confidence intervals reflect the between-study heterogeneity in the prognostic factor effects. The results of the figure are presented on the scale of log-cumulative hazard ratios, which is the scale of the linear predictor in our specifications. The prediction intervals for age and ${FEV_{1}}$ do not include 0 on the log-cumulative hazard scale, which indicates that a new study will find prognostic value of these variables with a probability of at least 0.95. Moreover, while the effect of ${FEV_{1}}$, appears to be fixed over time, the negative effect of age on the survival of COPD patients becomes more pronounced with time. By contrast, the predictive interval for the effect of the dyspnea score (mMRC) contains 0 at most of the time points presented in Figure 2B, which suggests that a larger proportion of future studies will not find relevant effects for this factor.

The posterior mode predictions of the random effects also give a sense of the between-study heterogeneity in the prognostic factor effects. These and the baseline log-cumulative hazard curves from Models 1 to 6 are presented in Section C of the Supplementary material available at *Biostatistics* online.

We validated the proposed models and the implementation by comparing our results to estimates from the flexible parametric PH model developed by Crowther *and others* (2014) and implemented in the $\textsf{Stata}$ package **merlin** (Crowther, 2020). Although the two approaches are formally very similar, there are important computational differences, which are summarized in Section A.2 of the Supplementary material available at *Biostatistics* online. The estimation of the mixed-effects specifications with **tramME** is 100+ times faster, emphasizing the importance of the developments presented herein, namely, using the Laplace approximation and automatic differentiation.

### 4.3. Internal–external validation of predictive performance

We finally assess the predictive performance of the proposed models. Our primary focus here is on ordering the different specifications (Models 1–6 in Table 1) based on how well their results can be generalized to future studies. For this reason, we adopt an internal–external validation strategy (Steyerberg and Harrell, 2016), splitting the sample by studies. In this approach, we essentially perform leave-one-out cross-validation on the level of studies, and calculate a measure of predictive performance by comparing predictions to the actual values in the omitted studies.

We compare the model specifications based on their *out-of-sample log-likelihood* values in a validation set. The out-of-sample log-likelihood (logarithmic score) is a proper scoring rule that takes the predictive distribution, not just point predictions, into account (Held and Sabanés Bové, 2014, Chapter 9). Unlike other traditional measures of discrimination and calibration, the out-of-sample log-likelihoods are easy to evaluate under various forms of (random) censoring.

A technical difficulty arises from the differences in the extent to which the various model specifications are applicable in a prognostic setting. In models expressing baseline risks proportional to a common baseline hazard and capturing differences in study-level baseline characteristics with normally distributed random intercepts (Models 1 and 3, in Table 1), predictive distributions of survival times can be calculated by integrating over the distribution of the random effects. In contrast, models with stratified baseline log-cumulative hazards by cohorts (Models 2, 4, 5, and 6) do not pool information on baseline risks across studies, and thus additional assumptions have to be introduced to make them suitable to predict absolute risks, that is, survival probabilities, for observations in a potentially new study. Debray *and others* (2013) and Steyerberg *and others* (2019) outline strategies for dealing with predictions in the case of stratified baseline risks. In our assessment of predictive performances, we re-estimate the baseline log-cumulative hazards from the IPD of the validation set. This way, our evaluation concentrates on the generalizability of the effects of the prognostic factors on individual predictions, and the uncertainties around them, as captured by the various models.

To make models with stratified and proportional random baseline risks comparable in an internal–external cross-validation scheme based on out-of-sample log likelihoods, we need to use auxiliary models that approximate the baseline risks in the validation set, while using the prognostic index estimates (Royston and Altman, 2013) from the estimation set. Formally, we separate the parameter vector ${\boldsymbol{\vartheta}}$ to the fixed effects parameters and the coefficients of the basis function that approximates the baseline risks: ${\boldsymbol{\vartheta}} = ({\boldsymbol{\vartheta}}_{0}^{\top}, {\boldsymbol{\beta}}^{\top})^{\top}$. We then estimate the parameters ${\boldsymbol{\beta}}^{(-i)}$ and ${\boldsymbol{\Sigma}}{}^{(-i)}$ by maximizing the logarithm of the likelihood, $\ell_{-i}({\boldsymbol{\vartheta}}_{0}^{(-i)}, {\boldsymbol{\beta}}^{(-i)}, {\boldsymbol{\Sigma}}{}^{(-i)})$,

$$\begin{eqnarray*}
\ell_{-i}({\boldsymbol{\vartheta}}_{0}^{(-i)}, {\boldsymbol{\beta}}^{(-i)}, {\boldsymbol{\Sigma}}{}^{(-i)}) =\sum_{\imath \in \{1, \dots, I\} \setminus i}\log\int_{{\boldsymbol{b}}_{\imath} \in
{\mathbb{R}}^{q}}{\boldsymbol{\vartheta}}_{\imath}\left(({{\boldsymbol{\vartheta}}_{0}^{(-i)}}^{\top}, {{\boldsymbol{\beta}}^{(-i)}}^{\top})^{\top}, {\boldsymbol{\Sigma}}{}^{(-i)}, {\boldsymbol{b}}_{\imath}\right)\mathsf{\,d}{\boldsymbol{b}}_{\imath},\end{eqnarray*}$$

described in (3.3), leaving the $i$th cohort out of the sample. In the next step, we fit an auxiliary model to the validation set by plugging in the maximum likelihood estimates of the prognostic factor effects and the random-effect covariance matrices, denoted by $\hat{\boldsymbol{\beta}}^{(-i)}$ and $\hat{\boldsymbol{\Sigma}}{}^{(-i)}$, respectively. Finally, to remove the effect of study-level differences in baseline risks, and make the measure comparable across cohorts, we center it with the value of the log-likelihood of an unconditional model, fitted to the validation set, ${\mathbb{P}}(T_{ij} \leq t_{ij}) = 1 -\exp\left[-\exp\left\{h_{i}\left(t_{ij}; {\boldsymbol{\vartheta}}_{0}^{(i)}\right) \right\} \right].$ Putting these elements together, the centered out-of-sample log-likelihood in the $i$th cohort is calculated as $\ell_{i}^{\text{C-OOS}}= \underset{{\boldsymbol{\vartheta}}_{0}^{(i)}}\sup\, \ell_{i}\left({\boldsymbol{\vartheta}}_{0}^{(i)},\hat{\boldsymbol{\beta}}^{(-i)}, \hat{\boldsymbol{\Sigma}}{}^{(-i)}\right) - \underset{{\boldsymbol{\vartheta}}_{0}^{(i)}}\sup\, \ell^{0}_{i}\left({\boldsymbol{\vartheta}}_{0}^{(i)}\right).$

Table 2 summarizes the centered log-likelihood values across the cohorts in the 3CIA data set. Based on these results, the most complex model (Model 4), that is, the model with stratified baseline risks, random slopes, and time-dependent fixed effects, has the best overall predictive performance. In general, the mixed-effects specifications resulted in higher out-of-sample log-likelihood values, compared to the fixed-effects only models. Moreover, stratification (Models 2 and 4) has led to improved predictive performance in our comparisons relative to the random intercepts alternatives. Finally, models that relax the PH assumption with time-varying effects tend to have slightly better predictive performance in the data set.

There seem to be substantial differences in the predictive performances of the various models, but it should be noted that it is hard to interpret these differences on the log-likelihood scale. Although there exist more traditional measures of predictive performance that are easier to interpret (most notably the $c$-index), the out of sample log-likelihood provides a proper alternative for *probability forecasts* (as opposed to point predictions) in the presence of interval-censoring and time-dependent effects. Moreover, the study-level predictive results are clearly not independent, hence the estimation of the variability around the aggregated out-of-sample log-likelihood values is complicated and beyond the scope of this study.

## 5. Simulation study

We conduct a simulation study to empirically evaluate our proposed model and the estimation method. Our goals are 2-fold: First, we confirm that our implementation of the mixed-effects transformation model is correct, and second, we compare the results from our approach to an alternative procedure introduced by Garcia *and others* (2019).

In this simulation exercise, we simulate new survival times from a model presented in Section 4. The time-varying PH specification of Model 3 contains 4D random effects, a frailty term to capture heterogeneities in the baseline risk across studies and three random slopes for the covariates. Departures from the PH assumptions are allowed in this model due to the time-varying mean prognostic factor effects. The conditional distribution function implied by the model can be written as

(5.7) \begin{align*} \small{\mathbb{P}}\left(T_{ij} \leq t_{ij} \mid {\boldsymbol{X}}_{ij} = {\boldsymbol{x}}_{ij},{\boldsymbol{\mathcal{B}}}^{\star}_{i} = (a_{i}, {\boldsymbol{b}}_{i}^{\top})^{\top}\right) &= 1 - \exp\left[-\exp\left\{h(t_{ij}) + a_{i} +{\boldsymbol{x}}_{ij}^{\top}({\boldsymbol{\beta}}(t_{ij}) + {\boldsymbol{b}}_{i}) \right\}\right], \label{eq:sim_mod} \end{align*}

with ${\boldsymbol{\mathcal{B}}}^{\star}_{i} \sim {\mathcal{N}}_{q+1}({\mathbf{0}}, {\boldsymbol{\Sigma}}{}^{\star})$. Because (5.7) is a fully specified probabilistic model, we can draw random samples by the golden rule.

Garcia *and others* (2019) present an estimation procedure for time-varying proportional odds mixed-effects models and apply it to perform IPD meta-analysis of clustered time-to-event data. Their approach is based on cutting the observations at distinct time points, and sequentially estimating binary outcome mixed-effects logistic regression models for each of these splits. As mentioned in the original article, by setting the link function to a complementary log–log transformation, instead of the logit function, we can easily modify the procedure to the time-varying PH model (5.7). Although the estimation can be done with standard software, the procedure is computationally demanding, because we have to estimate one binary outcome mixed-effects model for each time point at which we wish to evaluate the distribution function.

In the simulation we present below, we generate 500 random samples from the model defined by (5.7), using the data structure and covariate values of the 3CIA data set, as well as the estimated parameters we obtained in Section 4. We then re-estimate the mixed-effects transformation model and fit the time-varying PH mixed-effects model using the procedure by Garcia *and others* (2019) at the time points $t=30, 50, 90, 120, 150$ ($t$ denotes months in the original data set). For simplicity and fairness of comparison, we do not simulate censored observations and focus on exactly observed event times only.

**Fig. 3.**
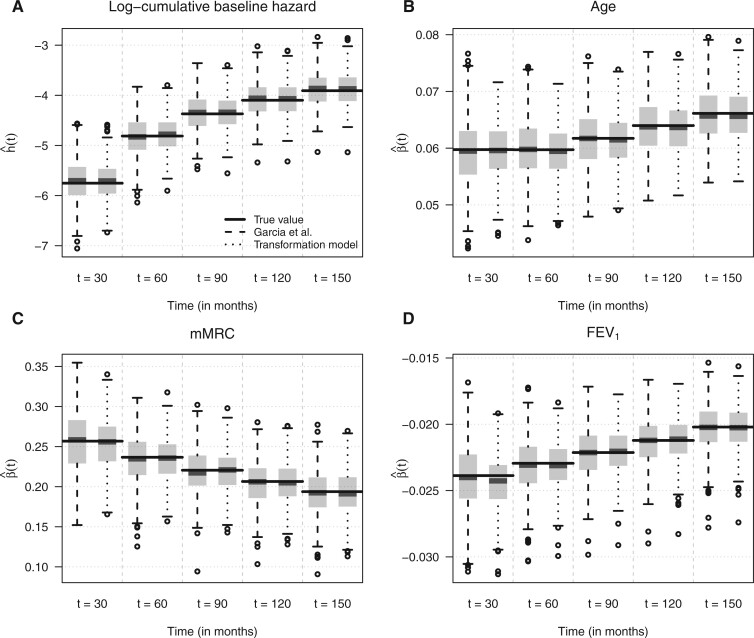
Comparison of the distribution of the time-varying fixed-effects estimates from the simulation study (500 iterations). (A) The log-cumulative baseline hazard is presented, while (B–D) depict the time-dependent conditional mean effects of the three covariates.

Figure 3 presents the distribution of the time-varying fixed effects estimates from the two approaches evaluated at five time points. As the results show, both methods give essentially unbiased estimates for the baseline log-cumulative hazard function as well as the time-dependent effects of the three covariates. Slightly smaller variabilities can be observed in the case of the mixed-effects transformation model, especially for smaller values of $t$. Further results of the simulation experiment (variance components, predictions for study-level effects) are presented in Section D of the Supplementary material available at *Biostatistics* online.

In summary, the results demonstrate that our implementation of mixed-effects transformation models in package **tramME** correctly recovers the parameters of the true model. A direct comparison with the estimation procedure by Garcia *and others* (2019) shows that the two approaches are comparable in terms of the estimated fixed effects (for the same individual time points) and the variance components. However, the simultaneous estimation procedure leads to slightly smaller variability, especially for smaller values of the outcome. The differences are more pronounced and show an advantage of the simultaneous estimation when we compare the predictions of the random effects. Moreover, the computational efforts of fitting the model simultaneously is about the same as estimating a mixed-effects regression with a dichotomous outcome at a *single* sample split. While fitting the mixed-effects transformation model required on average 40.65 s (median $=$ 39.25, 25th and 75th percentiles $=$ [34.42, 45.28], with a single nonconvergent case), the five model fits of the sample-splitting approach took 363.49 s (median $=$ 345.46, 25th and 75th percentiles $=$ [295.73, 409.27]). Consequently, our approach requires less computational resources and scales better, when we aim to evaluate the model at many values of the outcome.

We run a separate simulation experiment on the effect of misspecifying the inverse link function in a mixed-effects transformation model. Section D of the Supplementary material available at *Biostatistics* online presents results on the bias of the estimated covariate effects in the presence of a misspecified inverse link function.

## 6. Discussion

In this article, we proposed mixed-effects transformation models for the analysis clustered data and applied the method to IPD meta-analysis for time-to-event outcomes in a prognostic setting. We demonstrated that the model class is suitable for specifying and estimating model variants that are particularly useful in meta-analysis. Study-level heterogeneity is captured with general multi-dimensional random effects. Nonproportionality can be incorporated with the use of time-varying prognostic factor effects. Baseline patient characteristics can be modeled either by using stratified models or by introducing random frailty terms. Moreover, mixed-effects transformation models can be easily estimated in the presence of various forms of random censoring and truncation, which makes the approach attractive in many applied settings.

In the analysis of the 3CIA data set, we relied exclusively on the log-likelihood values (both in-sample and out-of-sample) as main tools of model selection. Other popular model selection criteria (AIC or BIC) are problematic in the case of mixed-effects transformation models because the parameter space is restricted to ensure the monotonicity of the conditional distribution function. In our experience, ignoring that the degrees of freedom are smaller than the number of parameters, for example, when choosing the polynomial orders, could lead to selecting the incorrect model. Developing tools for model selection in the class of transformation models is left for future research.

Nonlinear relationships between the outcome and the covariates are common, and several approaches have been suggested to model these in a IPD meta-analysis context (Sauerbrei and Royston, 2011; White *and others*, 2018). Our model could be extended to incorporate such effects for example by using basis transformations the same way we captured the baseline cumulative hazards or the time-varying effects (see Liu *and others*, 2017, for a similar approach). An interesting, although technically more challenging extension could be a model that includes nonlinear time-varying prognostic factor effects in the form of 2D smooths.

It should be emphasized that the potential applications of our proposed model go far beyond the IPD meta-analysis with survival outcomes presented in this article. In fact, mixed-effects transformation models can be estimated with any, at least ordered, outcomes. Moreover, due to the modular structure of the transformation model framework, our approach is easily modifiable and extensible for specific applications (for some examples, see Hothorn, 2020; Siegfried and Hothorn, 2020). The $\textsf{R}$ package for maximum likelihood estimation of mixed-effects transformation models (**tramME**, Tamási and Hothorn, 2021b) readily implements the most important functionality for IPD meta-analysis, with several other additions planned for the near future.

## Supplementary Material

kxab045_Supplementary_Data

## 7. Software

The software is available as an $\textsf{R}$ package (Tamási and Hothorn, 2021b), which contains a demo for estimating the models presented in this article. The $\textsf{R}$ code for replicating the simulation study and the $\textsf{Stata}$$\texttt{do}$ file for fitting the models with **merlin** (Crowther, 2020, version 1.2.0) is available at https://github.com/btamasi/tramme-ipd-ma.
