# Supplementary material for: Individual participant data meta-analysis with mixed-effects transformation models
Source: Biostatistics. 2021 Dec 30;23(4):1083–98. doi: 10.1093/biostatistics/kxab045 (PMC9566326; doi:10.1093/biostatistics/kxab045)
Supplement: kxab045_Supplementary_Data [file kxab045_supplementary_data.pdf]

Biostatistics (2021), **0**, 0, *pp.* 1-17
doi:10.1093/biostatistics/tramme-ipd-ma-supp

Supplementary material "Individual participant data meta-analysis with mixed-effects transformation models"

BÁLINT TAMÁSI

Institut für Epidemiologie, Biostatistik und Prävention, Universität Zürich, Switzerland

MICHAEL CROWTHER

Department of Medical Epidemiology and Biostatistics, Karolinska Institutet, Sweden

MILO ALAN PUHAN

Institut für Epidemiologie, Biostatistik und Prävention, Universität Zürich, Switzerland

EWOUT W. STEYERBERG

Department of Biomedical Data Sciences, Leiden University Medical Center, Netherlands

TORSTEN HOTHORN*

Institut für Epidemiologie, Biostatistik und Prävention, Universität Zürich, Hirschengraben 84, CH-8001 Zürich, Switzerland, `<Torsten.Hothorn@R-project.org>`

A. Computational details

A.1 *Transformation models with **tramME***

Following the approach described by Hothorn *and others* (2018), we approximate the baseline transformation functions, h_i in Equation (3.2), and the time-varying effects of the covariates,

*To whom correspondence should be addressed.

© The Author 2021. Published by Oxford University Press. All rights reserved. For permissions, please e-mail: journals.permissions@oup.com

$\boldsymbol{\beta}(t)$, by a polynomial in Bernstein form

$$\boldsymbol{a}_{\text{Bs},M}(t)^\top \boldsymbol{\vartheta}_0 = \frac{1}{M+1}\sum_{m=0}^{M} \vartheta_{0,m}\binom{M}{m}\tilde{t}^m(1-\tilde{t})^{M-m},$$

where the elements of $\boldsymbol{a}_{\text{Bs},M}(t)$ form a *Bernstein basis* of order M (Farouki, 2012) and $\tilde{t}$ is the rescaled version of t to the support $[0,1]$. The choice of the order of these polynomials is extensively discussed in Hothorn (2020). In the application presented in this text, the results are very similar from models with polynomial order above four, so we used this order for all six model variants.

The integral in the log-likelihood function (see Equation 3.6 of the article) is evaluated using the Laplace approximation. The Laplace approximation is based on the second order Taylor expansion of the joint log-likelihood function at the conditional mode of the random effects, $\hat{\boldsymbol{b}}_i(\boldsymbol{\vartheta},\boldsymbol{\Sigma}) = \arg\max_{\boldsymbol{b}_i} \mathcal{L}_i(\boldsymbol{\vartheta},\boldsymbol{\Sigma},\boldsymbol{b}_i)$. In the context of IPD meta-analysis, these random effects predictions are the deviations of the individual studies from the mean effect sizes. The approximation of the log-likelihood function is

$$\ell^{\text{LA}}(\boldsymbol{\vartheta},\boldsymbol{\Sigma}) = \sum_{i=1}^{I}\left[\log \mathcal{L}_i\left(\boldsymbol{\vartheta},\boldsymbol{\Sigma},\hat{\boldsymbol{b}}_i(\boldsymbol{\vartheta},\boldsymbol{\Sigma})\right) - \frac{1}{2}\log|\boldsymbol{H}_i(\boldsymbol{\vartheta},\boldsymbol{\Sigma})| + n_i\log(\sqrt{2\pi})\right],$$

with

$$\boldsymbol{H}_i(\boldsymbol{\vartheta},\boldsymbol{\Sigma}) = -\left.\frac{\partial^2 \log \mathcal{L}_i(\boldsymbol{\vartheta},\boldsymbol{\Sigma},\boldsymbol{b}_i)}{\partial \boldsymbol{b}_i^2}\right|_{\boldsymbol{b}_i=\hat{\boldsymbol{b}}_i(\boldsymbol{\vartheta},\boldsymbol{\Sigma})}.$$

In order to improve numerical properties of the models estimated in example application, we rescaled the covariates using their ranges. This way, convergence for the otherwise complicated models was more easily attained, and the results became more stable. Wherever it was necessary, the results were cast back to their original scale. The corresponding standard errors were recalculated using the delta method.

The maximum likelihood estimation of mixed-effects transformation models is implemented in the R add-on package **tramME** (Tamási and Hothorn, 2021), and the fixed-effects transformation

models are estimated using the **tram** and **mlt** packages (Hothorn, 2020). For the implementation details and several worked example applications, see the package vignettes.

The model specifications described in Table 1 and used in our example application in Section 4 can be estimated with the following commands.

```
R> library("tram")
R> library("tramME")

R> sup <- c(1, 150) ## setting the support for basis approximation
R> opt <- optim_control(iter.max = 1e4, eval.max = 1e4, rel.tol = 1e-8)

R> ## ====== MODEL 1
R> fit_m1 <- CoxphME(sui ~ ages + mmrcs + fev1pps + (ages + mmrcs + fev1pps | cohort),
+    data = dat, log_first = TRUE, order = 4, support = sup,
+    control = opt)

R> ## ====== MODEL 2
R> fit_m2 <- CoxphME(
+    sui | 0 + cohort ~ ages + mmrcs + fev1pps + (0 + ages + mmrcs + fev1pps | cohort),
+    data = dat, log_first = TRUE, order = 4, support = sup,
+    control = opt)

R> ## ====== MODEL 3
R> fit_m3 <- CoxphME(
+    sui | ages + mmrcs + fev1pps ~ 1 + (ages + mmrcs + fev1pps | cohort),
+    data = dat, log_first = TRUE, order = 4, support = sup,
+    control = opt)

R> ## ====== MODEL 4
R> fit_m4 <- CoxphME(
+    sui | 0 + cohort + ages + mmrcs + fev1pps ~ 1 +
+      (0 + ages + mmrcs + fev1pps | cohort),
+    data = dat, log_first = TRUE, order = 4, support = sup,
+    control = opt)

R> ## ====== MODEL 5
R> fit_m5 <- Coxph(sui | 0 + cohort ~ ages + mmrcs + fev1pps, data = dat,
+    log_first = TRUE, order = 4, support = sup)

R> ## ====== MODEL 6
R> fit_m6 <- Coxph(sui | 0 + cohort + ages + mmrcs + fev1pps ~ 1, data = dat,
+    log_first = TRUE, order = 4, support = sup)
```

The estimation on a subset of the original 3CIA sample can be performed by running the demo “IPD-MA” of the **tramME** package:

```
R> demo("IPD-MA", package = "tramME")
```

A.2 *Comparison of **tramME** and **merlin***

To further validate the proposed models and implementation, we have also replicated the core results in Stata using the mixed-effects model developed by Crowther *and others* (2014), and implemented in the **merlin** package (Crowther, 2020). **merlin** fits a wide range of multivariate multilevel regressions, of which the mixed-effects Royston-Parmar model is a special case. Similarly to the transformation models proposed in the main text, the model by Royston and Parmar (2002) parameterizes the log-cumulative hazards.

Although the two models are formally very similar, there are several important computational differences between the two implementations. Table S-1 gives a summary of these. **merlin** uses the original Royston-Parmar formulation and utilizes natural cubic splines to approximate the baseline log-cumulative hazards *without* introducing explicit constraints to ensure the monotonicity of the function (see Royston and Parmar, 2002, pp. 2177). **merlin** estimates the fixed effects only, univariate models with analytic gradients, and hence fitting Models 5 and 6 are computationally highly efficient. However, models with random effects firstly use adaptive Gauss-Hermite quadrature to calculate the likelihood, and secondly use finite differences for all gradient calculations. In contrast, **tramME** uses polynomials in Bernstein form with corresponding explicit constraints on their parameters to capture the baseline log-cumulative hazard and the time-varying effects. Building on the automatic differentiation and Laplace approximation provided by the **TMB** package (Kristensen *and others*, 2016), the estimation of the presented models with **tramME** is very efficient. The slower computations render the fitting of the mixed-effects models with **merlin** to the 3CIA dataset practically infeasible.

The Stata code for estimating the six model variants with the **merlin** package is available from Github at `https://github.com/btamasi/tramme-ipd-ma`.

We compare the numerical results from the fitting Model 5 (homogeneous, time-invariant effects of the predictors, stratified baseline risk) with both **tramME** and **merlin** to verify the

Table S-1. Comparison of the computational details of mixed-effects models in the R package **tramME** (Tamási and Hothorn, 2021) and the Stata package **merlin** (Crowther, 2020).

	**tramME**	**merlin**
Transformation scale	Log-cumulative hazard	Log-cumulative hazard
Parametrization	Bernstein basis	Natural cubic spline basis
Parameter restrictions	Explicit	Implicit
Gradient calculations	Automatic differentiation	Finite differences
Numerical integration of the log-likelihood	Laplace approximation	Gauss-Hermite quadrature

validity of our implementation. To facilitate the reproducibility of these calculations, we used the anonymized subset of the original 3CIA dataset that we made available as part of a demo in the R package **tramME**. Table S-2 shows that the parameter estimates and log-likelihood values from the two packages are very similar.

Table S-2. Comparison of estimation results of Model 5 using **tramME** and **merlin**. Coefficient estimates are denoted by the names of the covariates. Standard errors are shown in parentheses.

	**tramME**	**merlin**
Age	0.061 (0.002)	0.062 (0.002)
mMRC	0.184 (0.019)	0.187 (0.019)
FEV_1	-0.017 (0.001)	-0.017 (0.001)
$\log \mathcal{L}$	-15746.81	-15837.09

The estimation of Model 6 with the two implementations on the smaller dataset also gives similar results. We only report the log-likelihood values here, which are $-15\,774.44$ and $-15\,730.49$ in the case of **merlin** and **tramME**, respectively.

B. The 3CIA dataset

Figure S-1 shows the distributions of the outcome variable (time to death) and the three prognostic factors (age, FEV_1, and the modified Medical Research Council (mMRC) dyspnea score).

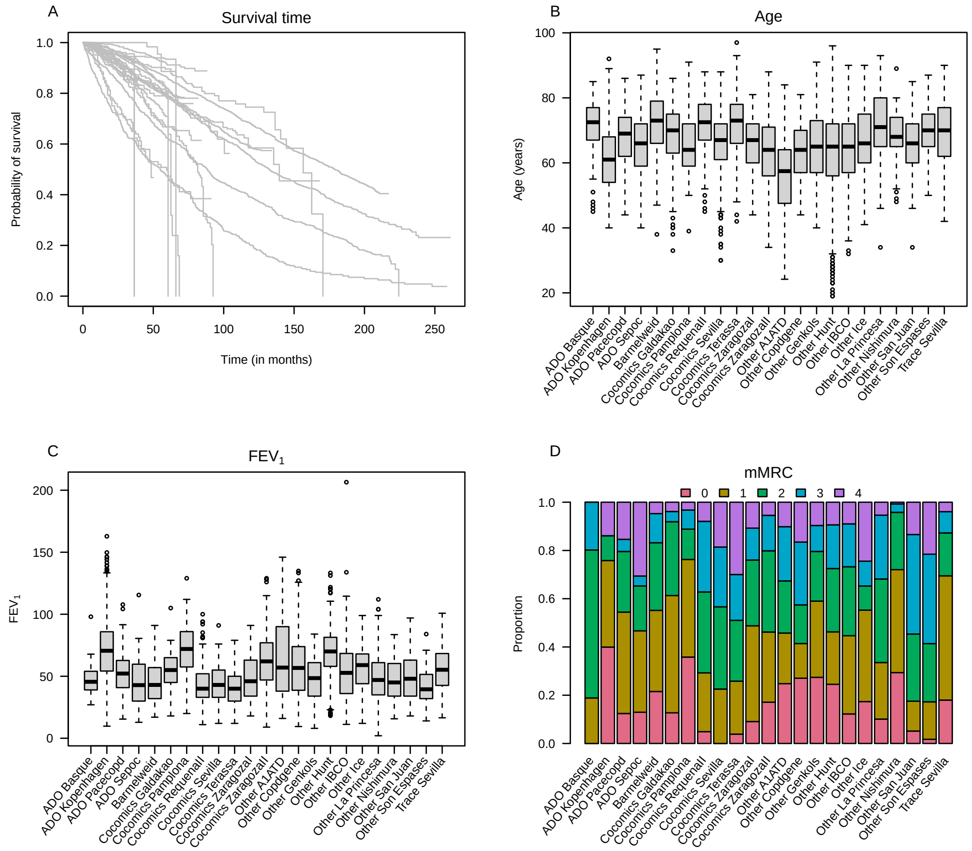

Fig. S-1. Distribution of survival times (panel A) and prognostic factors (panels B, C and D) by cohorts of the 3CIA dataset.

B.1 *Censoring*

The outcome variable, the survival time of the participants, is recorded with different precision in different cohorts. In most of the cases, the follow-up time is rounded to months, while in other cohorts the same information is available in fractions of months. It is clear, that having only rounded follow-up times represents partial information on the actual times of events, and a correct statistical approach must take this issue into account. For this reason, we treat the observations recorded with follow-up times rounded to zero and one decimal places as *interval-censored* observations, while survival times with two decimal places as exact observations. The likelihood

contributions of exact continuous and interval-censored observations are given by Equation (3.4) and by the third case in Equation (3.5) of the article, respectively. Our proposed estimation procedure can easily accommodate both types of observations.

The distribution of censoring times for each cohort is given by Figure S-2. Because there are considerable differences in the lengths of the various studies, the presence of administrative censoring times makes it difficult to find any patterns in the censoring distributions of the dataset. Some studies exhibit only very late censorings, presumably due to administrative censoring. In other studies, censoring started early and occured over a longer period, maybe due to loss-of-follow-up.

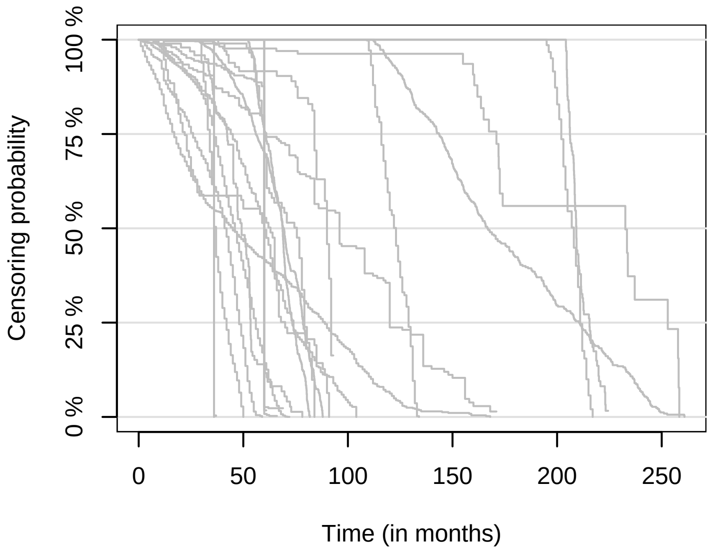

Fig. S-2. Distribution of censoring times in the various cohorts.

B.2 *Missing data*

In our analysis, we only consider patients with complete information on the outcome and covariates to avoid additional technical complications and to focus on presenting the proposed model class. For this reason, we remove all individuals that had missing values in either the outcome variable (the length of the follow-up and the status at the end of follow-up) or any of the three prognostic factors.

Figure S-3 presents the proportions of missing values in the various studies. Due to systematic missingness, that is, when one of the prognostic factors were not recorded in a cohort, we had to discard two complete, albeit small, cohorts ("Cocomics Tenerife", 276 subjects and "Cocomics Requena I", 173 subjects).

Our choice of focusing on the complete cases only is motivated by the fact that the proportion of missing observations is relatively small and our primary goal is to demonstrate the use of the proposed model in the context of IPD meta-analysis without introducing any further statistical and numerical complexities. The mixed-effects transformation approach can, of course, be applied in conjunction with any standard imputation techniques.

B.3 *Between-study heterogeneity*

Table S-3 presents summary measures of the between-study heterogeneity in the case-mix in the 3CIA dataset. The quantiles of the outcome variable show substantial differences in the survival distributions in the various cohorts, which also can be seen in panel A of Figure S-1. Note that some cohorts do not contain enough number of events to calculate the medians and the 95th percentiles using the non-parametric maximum likelihood estimator.

Panels B, C and D of Figure S-1 indicate considerable heterogeneity in the distributions of individual predictors. Summary measures of the distribution of the patient characteristics can be obtained from the linear predictor values of the "global" fixed-effects only Model 5 (with

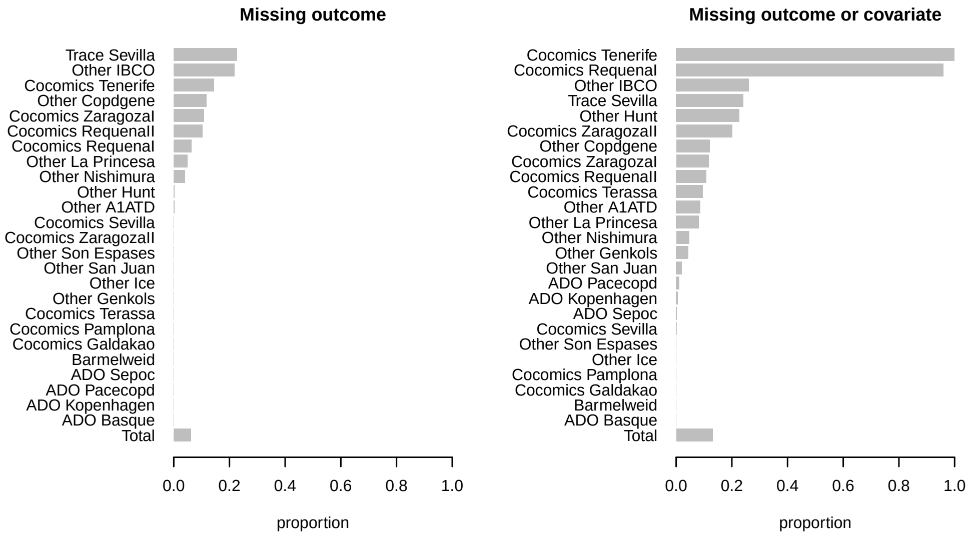

Fig. S-3. The proportion of missing outcome information (left) and response or covariate values (right) in the cohorts.

stratified baseline risks, Table 1). In this case, the mean and standard deviation of the linear predictor describe the location and the variability of a weighted sum of the prognostic factors in each cohort. As Table S-3 shows, there are differences in both the location of the linear predictor and its variability across studies.

To gauge the level of heterogeneity in the prognostic factor effects as well as the baseline risks, we first consider the "fully stratified model", i.e., fitting a Cox proportional hazards model with a fully parametric baseline cumulative hazard to each cohort separately. This approach corresponds to the first step of a two-stage IPD meta-analysis using a specification analogous to Model 2 (see Table 1). Fitting separate models to each cohort is considered suboptimal, because the estimates for the different studies do not borrow information from others, but it is a natural first step in assessing the heterogeneity in the prognostic factor effects, as it does not introduce too many additional assumptions. For simplicity, we only estimate the model with time-constant effects. Figure S-4 presents the results of the cohort-by-cohort estimation of the prognostic factor effects,

Table S-3. Between-study heterogeneity in the case-mix: Quantiles of the outcome variable calculated using the non-parametric maximum likelihood (Turnbull) estimator, and the mean and standard deviation of the linear predictor in the fixed-effects only, stratified proportional hazards model.

	Time-to-death quantiles			Linear predictor		
Cohort	5%	50%	95%	mean	SD	n_i
ADO Basque	18.50	—	—	3.91	0.69	106
ADO Kopenhagen	26.50	36.50	36.50	2.57	0.96	2273
ADO Pacecopd	20.65	—	—	3.56	0.74	338
ADO Sepoc	11.50	36.50	36.50	3.62	0.91	317
Barmelweid	6.50	48.50	—	3.96	0.77	232
Cocomics Galdakao	13.50	62.50	68.50	3.47	0.68	543
Cocomics Pamplona	36.50	156.50	170.50	2.84	0.91	215
Cocomics RequenaII	10.50	59.50	—	4.03	0.73	164
Cocomics Sevilla	6.95	88.35	224.35	3.76	0.74	581
Cocomics Terassa	3.35	54.85	228.45	4.23	0.71	337
Cocomics ZaragozaI	22.50	81.50	92.50	3.53	0.72	121
Cocomics ZaragozaII	16.32	132.48	—	3.03	0.89	918
Other A1ATD	46.55	—	—	2.50	1.20	472
Other Copdgene	24.50	—	—	3.20	0.91	3945
Other Genkols	17.50	—	—	3.42	0.93	912
Other Hunt	30.50	178.50	—	2.86	1.06	1215
Other IBCO	20.50	139.50	—	3.34	0.91	1046
Other Ice	25.25	66.05	66.05	3.45	0.87	409
Other La Princesa	27.50	—	—	3.94	0.84	465
Other Nishimura	13.50	60.50	60.50	3.63	0.63	143
Other San Juan	55.65	—	—	3.66	0.81	97
Other Son Espases	14.45	—	—	4.11	0.56	116
Trace Sevilla	14.50	—	—	3.47	0.85	534

which are interpreted as log-hazard ratios in our model specifications.

Figure S-5 depicts the study-level baseline log-cumulative hazard functions. The individual cohorts differ in the baseline risks and the proportionality assumption among the baseline hazards (see Models 1 and 3 of Table 1) is violated, as some of the baseline transformation functions cross.

C. Additional results from model estimation

This section presents some additional results from the IPD meta-analysis of the 3CIA dataset using transformation models.

Figure S-6 compares the predicted values (posterior modes) of the random slopes for each

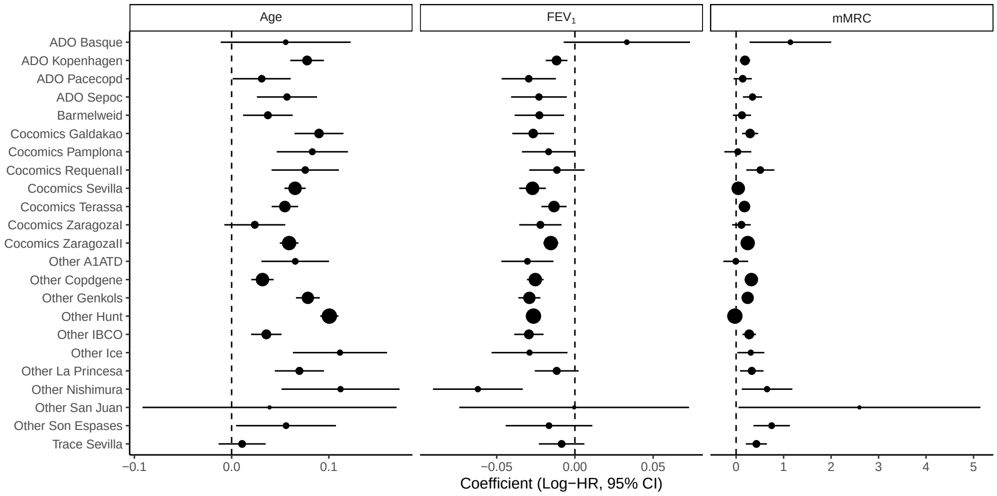

Fig. S-4. Coefficients and their 95% CIs of prognostic factors estimated for each study separately. The estimates are interpreted as log-hazard ratios. The sizes of the points are proportional to the number of events in the studies.

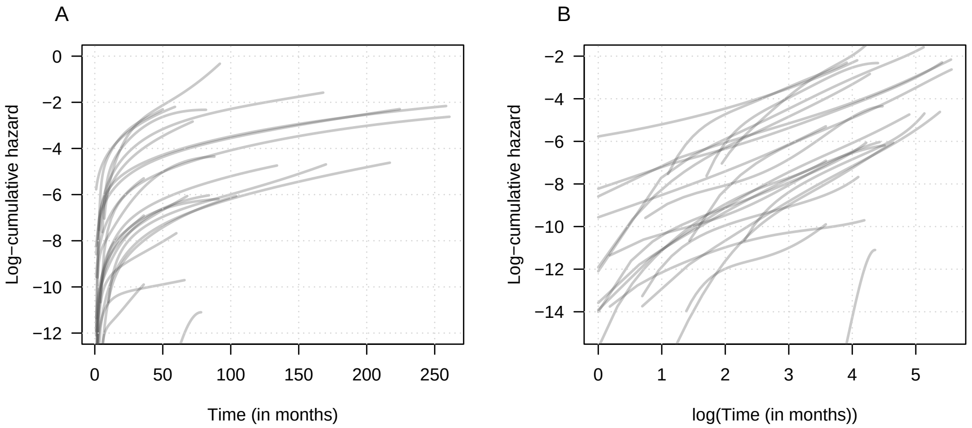

Fig. S-5. Baseline log-cumulative hazards from the fully stratified model, plotted against time (panel A) and log-time (panel B), respectively.

cohort in the 3CIA dataset from the mixed-effects model specifications (Models 1 to 4, in Table 1).

Figure S-7 presents the baseline transformation functions, (log-cumulative baseline hazards) estimated using Models 1 to 6 (see Table 1) for each study in the 3CIA dataset. Note that Models 1 and 3 assume proportional baseline hazards, which is reflected in the fitted curves in this plot.

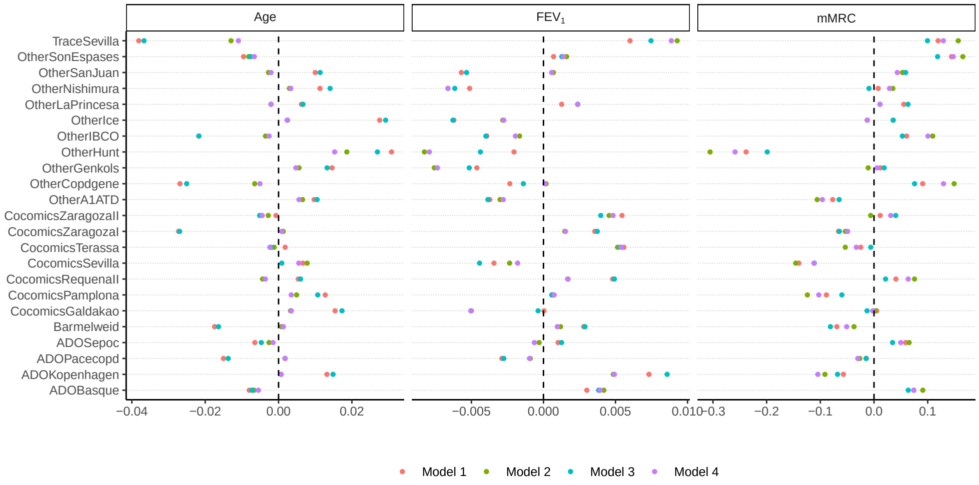

Fig. S-6. Predicted values of the random slopes in the mixed-effects transformation models.

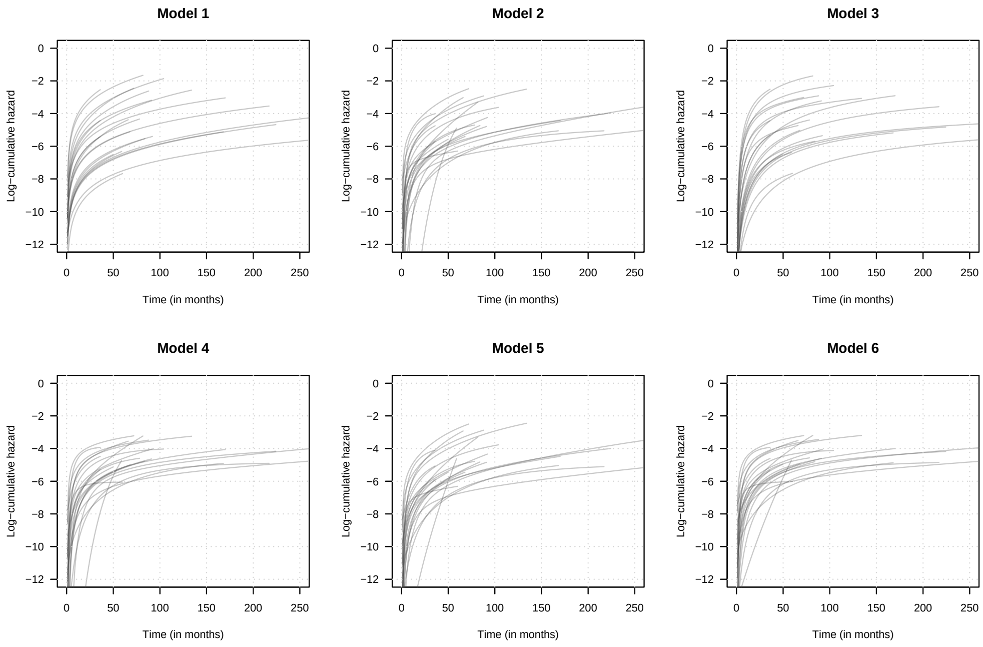

Fig. S-7. Baseline transformation functions of the cohorts in the 3CIA dataset estimated with different models.

D. Additional simulation results

In this section, we discuss further results of the simulation study that compares the mixed-effects transformation model approach for IPD-MA with the results obtained from the procedure

outlined in Garcia *and others* (2019).

The estimation of the variance components parameters can be evaluated based on Figure S-8. We generated a four-dimensional random-effects vector for each study in the dataset (random frailty term and three random slopes). Because the procedure by Garcia *and others* (2019) consists of re-estimating binary mixed-effects models at each time point, at which we want to evaluate the distribution of the outcome, we obtain five sets of random-effects covariance parameters, which we present separately. Similarly to our previous results, both of the approaches fare well in the specific estimation task, with the mixed-effects transformation model having slightly smaller variances. Slight downward bias can be observed in the variance estimates of the random effects with both approaches, which is in line with previous findings about maximum likelihood estimates of variance parameters in a mixed-effects IPD meta-analysis setting (Crowther *and others*, 2012). We also compare the models by their ability to predict the true values of the random effects. As Figure S-9 shows, in terms of root-mean-square errors (RMSE), the mixed-effects transformation model gives better predictions of the individual deviances from the baseline risks and covariate effects of the separate studies. In these comparisons, we pool the five posterior mode estimates generated by the procedure by Garcia *and others* (2019).

To evaluate the effect of misspecifying the inverse link function in a mixed-effects transformation model, we run a separate simulation experiment, which uses the data generating mechanism that corresponds to Model 1, with parameter values estimated from the 3CIA dataset. Instead of fitting the correct model, we fit a specification that uses expit $=$ logit^{-1}, the CDF of the standard logistic regression, as the inverse link function:

$$\mathbb{P}\left(T_{ij} \leqslant t_{ij} \mid \boldsymbol{X}_{ij} = \boldsymbol{x}_{ij}, \boldsymbol{\mathcal{B}}_i^{\star} = (a_i, \boldsymbol{b}_i^{\top})^{\top}\right) = \operatorname{expit}\left(h(t_{ij}) + a_i + \boldsymbol{x}_{ij}^{\top}(\boldsymbol{\beta} + \boldsymbol{b}_i)\right),$$
$$\boldsymbol{\mathcal{B}}_i^{\star} \sim \mathcal{N}_{q+1}(\mathbf{0}, \boldsymbol{\Sigma}^{\star}).$$

This specification corresponds to a *proportional odds* (PO) models. To compare the results with the correctly specified model, as well as the true effects, we transform the ef-

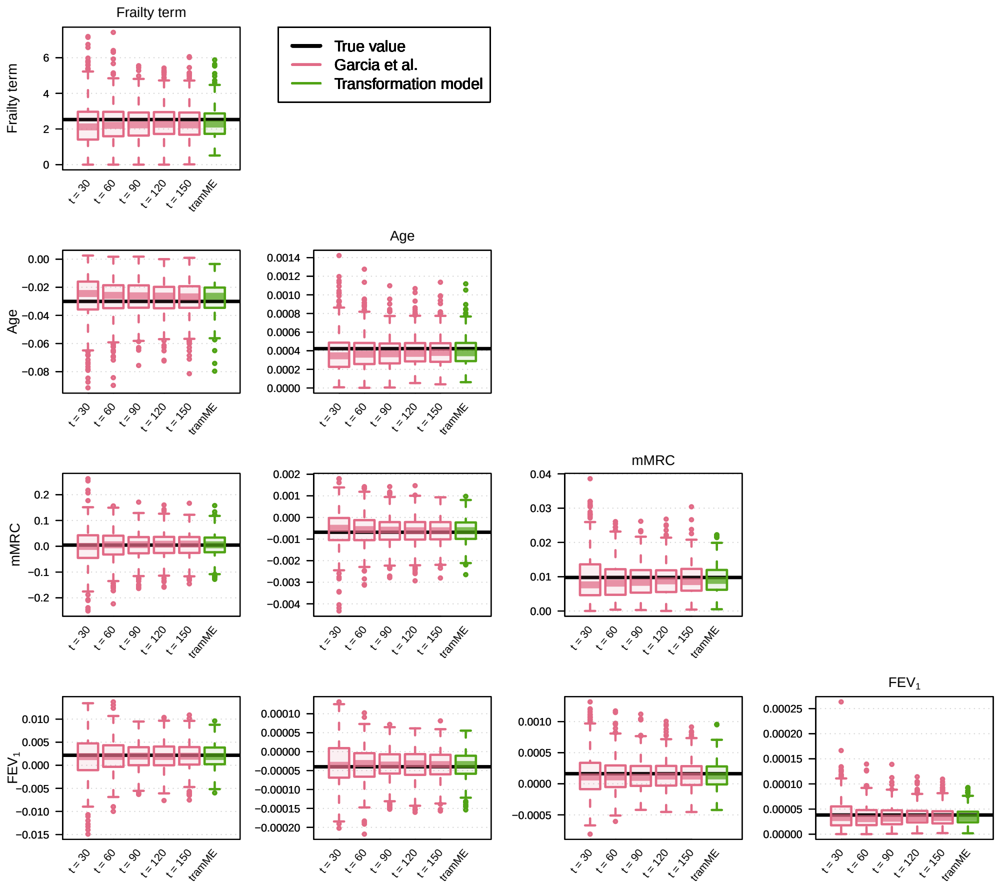

Fig. S-8. Distribution of the estimations of the random-effects covariance parameters from the simulation study. Each panel represents a parameter in the same position as in the covariance matrix of the data generation mechanism.

fect estimates from both models to the probability scale and calculate *probabilistic indices*, $\mathbb{P}\left(T \leqslant T' \mid \boldsymbol{X} = \boldsymbol{x}, \boldsymbol{X}' = \boldsymbol{x}', \boldsymbol{\mathcal{B}}^{\star} = \boldsymbol{b}^{\star}\right)$ (Thas *and others*, 2012). The probabilistic index (PI) gives the probability that an individual with a vector of covariate values ($\boldsymbol{x}$) has shorter survival time than an other individual in the same study that has the same predictor values except for one, which is larger by one unit ($\boldsymbol{x}'$). By integrating over the distribution of the corresponding random effects, we can calculate the expected PI that will serve as a common scale to compare our

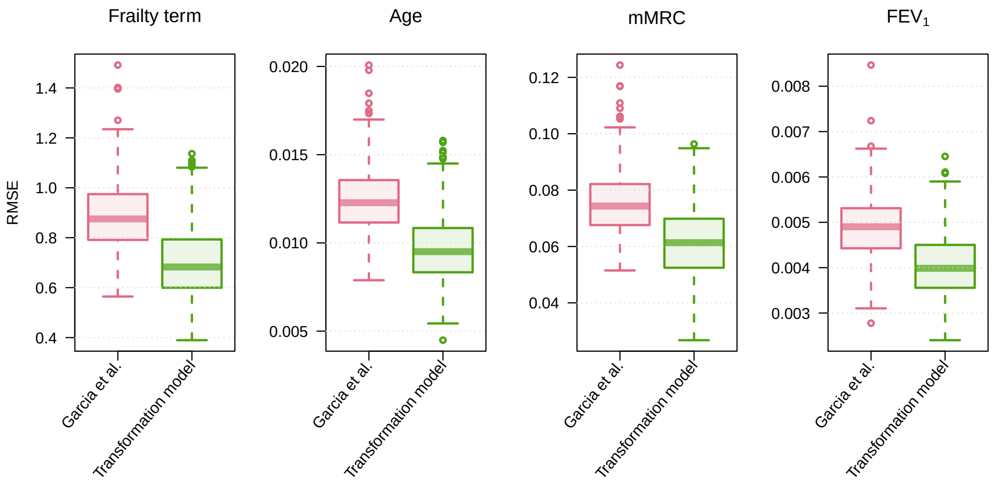

Fig. S-9. The distribution of the root-mean-square errors of the random-effects predictions from the two approaches in the simulation study. In the case of Garcia *and others* (2019), we pooled the five sets of predictions.

coefficients estimated from the PH and PO models:

$$\mathrm{PI} = \int_{-\infty}^{\infty} \mathbb{P}\left(T \leqslant T' \mid \boldsymbol{X} = \boldsymbol{x}, \boldsymbol{X}' = \boldsymbol{x}', \boldsymbol{\mathcal{B}}^{\star} = \boldsymbol{b}^{\star}\right) \phi(\boldsymbol{b}^{\star})\, \mathsf{d}\boldsymbol{b}^{\star}.$$

This transformation can be calculated for both the PH model and the PO model, because both transformation models are fully specified probabilistic models.

The effect estimates from the correctly specified model and the proportional-odds variant are compared in Figure S-10. The model that uses the correct inverse link function (PH) gives unbiased estimates of the effects, while the estimates of the misspecified model (PO) are biased on the probability scale.

Likelihood-based comparisons can aid the choice of the inverse link function. In our simulation, the log-likelihood values of the correctly specified model were larger in all simulation runs. The out-of-sample log-likelihood values, on an independent dataset of the same structure as the estimation sample, were also higher in every iteration when the inverse link was correctly specified.

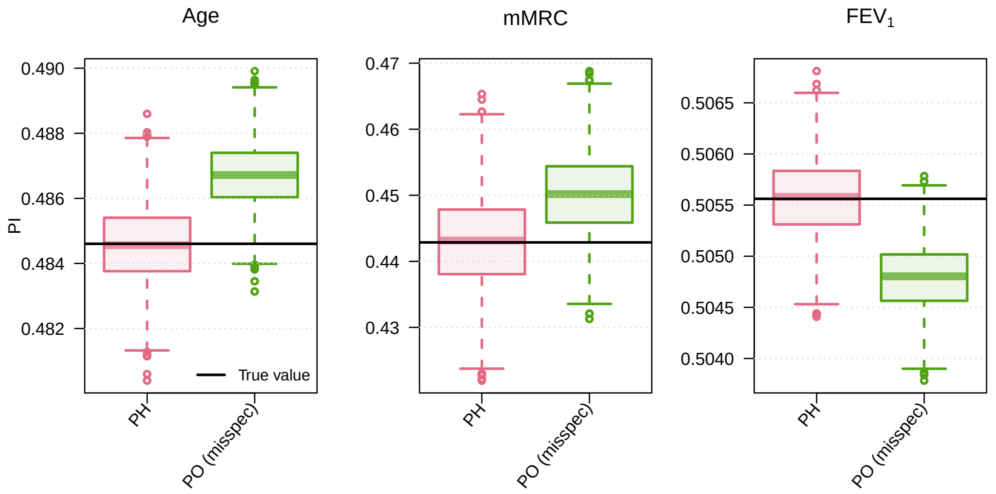

Fig. S-10. The effect of misspecifying the inverse link function. Comparison of probabilistic index (PI) estimates from proportional hazards (PH, correct specification) and proportional odds (PO, misspecified inverse link). The boxplots compare the distribution of 1000 draws to the true value based on Model 1.

References

Crowther, Michael J. (2020). merlin–A unified modeling framework for data analysis and methods development in Stata. *The Stata Journal* **20**(4), 763–784.

Crowther, Michael J., Look, Maxime P. and Riley, Richard D. (2014). Multilevel mixed effects parametric survival models using adaptive Gauss–Hermite quadrature with application to recurrent events and individual participant data meta-analysis. *Statistics in Medicine* **33**(22), 3844–3858.

Crowther, Michael J., Riley, Richard D., Staessen, Jan A., Wang, Jiguang, Gueyffier, Francois and Lambert, Paul C. (2012). Individual patient data meta-analysis of survival data using Poisson regression models. *BMC Medical Research Methodology* **12**(1), 34.

Farouki, Rida T. (2012). The Bernstein polynomial basis: A centennial retrospective. *Computer*

Aided Geometric Design **29**(6), 379–419.

Garcia, Tanya P., Marder, Karen and Wang, Yuanjia. (2019). Time-varying proportional odds model for mega-analysis of clustered event times. *Biostatistics* **20**(1), 129–146.

Hothorn, Torsten. (2020). Most likely transformations: The mlt package. *Journal of Statistical Software* **92**(1), 1–68.

Hothorn, Torsten, Möst, Lisa and Bühlmann, Peter. (2018). Most likely transformations. *Scandinavian Journal of Statistics* **45**(1), 110–134.

Kristensen, Kasper, Nielsen, Anders, Berg, Casper W., Skaug, Hans J. and Bell, Bradley M. (2016). TMB: Automatic differentiation and Laplace approximation. *Journal of Statistical Software* **70**(5), 1–21.

Royston, Patrick and Parmar, Mahesh K. B. (2002). Flexible parametric proportional-hazards and proportional-odds models for censored survival data, with application to prognostic modelling and estimation of treatment effects. *Statistics in Medicine* **21**(15), 2175–2197.

Tamási, Bálint and Hothorn, Torsten. (2021*a*). tramME: Mixed-effects transformation models using Template Model Builder. *The R Journal*. forthcoming.

Tamási, Bálint and Hothorn, Torsten. (2021*b*). *tramME: Transformation Models with Mixed Effects*. R package version 0.1.2, `https://CRAN.R-project.org/package=tramME`.

Thas, Olivier, Neve, Jan De, Clement, Lieven and Ottoy, Jean-Pierre. (2012). Probabilistic index models. *Journal of the Royal Statistical Society: Series B (Statistical Methodology)* **74**(4), 623–671.

[*Submitted April 21, 2021; revised October 25, 2021*]
